# Correction and Republication: Early Estimates of Bivalent mRNA Vaccine Effectiveness in Preventing COVID-19–Associated Emergency Department or Urgent Care Encounters and Hospitalizations Among Immunocompetent Adults — VISION Network, Nine States, September–November 2022

**DOI:** 10.15585/mmwr.mm7211a6

**Published:** 2023-03-17

On December 16, 2022, *MMWR* published “Early Estimates of Bivalent mRNA Vaccine Effectiveness in Preventing COVID-19–Associated Emergency Department or Urgent Care Encounters and Hospitalizations Among Immunocompetent Adults — VISION Network, Nine States, September–November 2022” (*1*). On January 25, 2023, one of the sites contributing data to the analysis notified CDC co-authors about an error in reporting history of receipt of bivalent doses. *MMWR* was notified about these concerns on February 10, 2023. The site corrected its vaccination history reporting, and the authors have corrected the report and confirmed that the reporting issue did not change the interpretation or the conclusions of the original report. In accordance with December 2017 guidance from the International Committee of Medical Journal Editors (*2*), *MMWR* is republishing the report (*3*). The republished report includes the original report with clearly marked corrections as supplementary materials.
